# Vitiligo overlying varicose veins: Three cases of a rare possible Koebner phenomenon

**DOI:** 10.1016/j.jdcr.2023.11.020

**Published:** 2023-12-04

**Authors:** Stephanie A. Matthews, Felipe B. Cerci, Stacy J. Echeverria Quintana, Caio C. Silva de Castro, Stanislav N. Tolkachjov

**Affiliations:** a School of Medicine, University of Kansas Medical Center, Kansas City, Kansas; b Dermatology Service, Hospital Universitário Evangélico Mackenzie, Curitiba, Brazil; c Post-graduate Program – Internal Medicine and Health Sciences, Universidade Federal do Paraná, Curitiba, Brazil; d Clínica Cepelle, Curitiba, Brazil; e Division of Dermatology, Hospital de Dermatologia Sanitária do Paraná, Curitiba, Brazil; f Pontifícia Universidade Católica do Paraná, Curitiba, Brazil; g Epiphany Dermatology, Dallas, Texas; h Department of Dermatology, The University of Texas at Southwestern Medical Center, Dallas, Texas; i Division of Dermatology, Baylor Scott & White, Dallas, Texas; j Division of Dermatology, Texas A&M College of Medicine, Dallas, Texas

**Keywords:** autoimmune, Koebner phenomenon, polypodium leucotomos, supplements, varicose vein, vascular, vitiligo

## Introduction

The Koebner phenomenon (KP) is characterized by the development of isomorphic lesions in traumatized but previously uninvolved skin.^1^, ^2^, ^3^ In generalized vitiligo, KP may affect more than one-third of patients and may be induced by various forms of trauma.^4^ To our knowledge, this is the third report of vitiligo affecting the skin over a varicose vein. Herein, we report 3 rare cases of vitiligo following the path of a varix, which may be attributed to the KP. The presence of koebnerization can have important implications for treatment response and disease severity,^5^ highlighting the importance for dermatologists to recognize rare presentations of the KP in vitiligo patients.

## Case report

**Fig 1:**
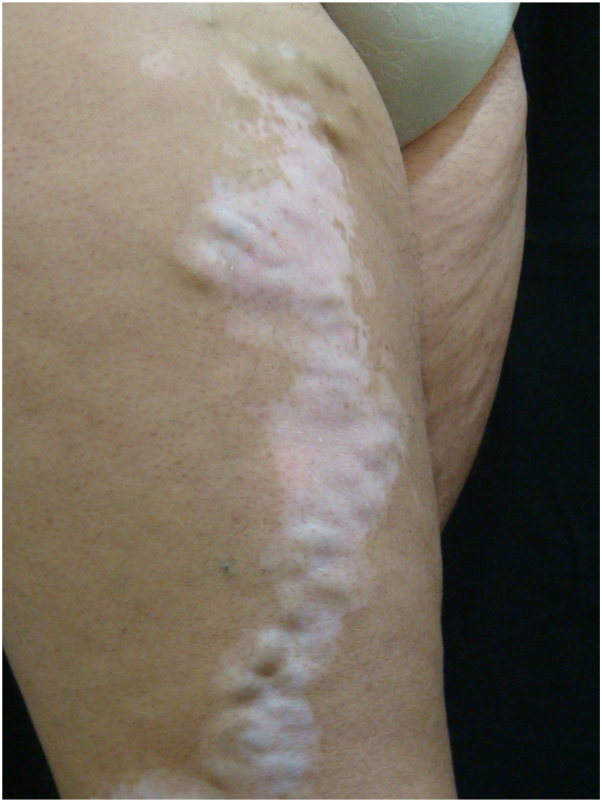
Linear patch of vitiligo overlying the dilated varix on the ventral and lateral thigh.

Case 1: A 58-year-old woman presented to the dermatology department with a history of asymptomatic depigmented macules and patches. The lesions began on her right posterior leg, followed the course of a varix, and progressively increased in size and number over the previous 20 years. New lesions of similar appearance had also appeared after minor trauma to other locations on her skin. Past medical history and family history included hypothyroidism, and 3 cousins with vitiligo, respectively. On physical examination, symmetric depigmented patches were distributed on her hands, wrists, axilla, and trunk. Asymmetric depigmentation was located on the lower limbs. On her right lower limb, 1 patch followed the course of a saphenous varix and was confined to this area (Fig 1). She denied any history of trauma to her right lower extremity or experiencing any pain or discomfort at this location. A clinical diagnosis of generalized vitiligo with KP secondary to a varicose vein was made. Topical corticosteroids, topical tacrolimus, and over 200 sessions of phototherapy have not shown any clinical improvement.

**Fig 2:**
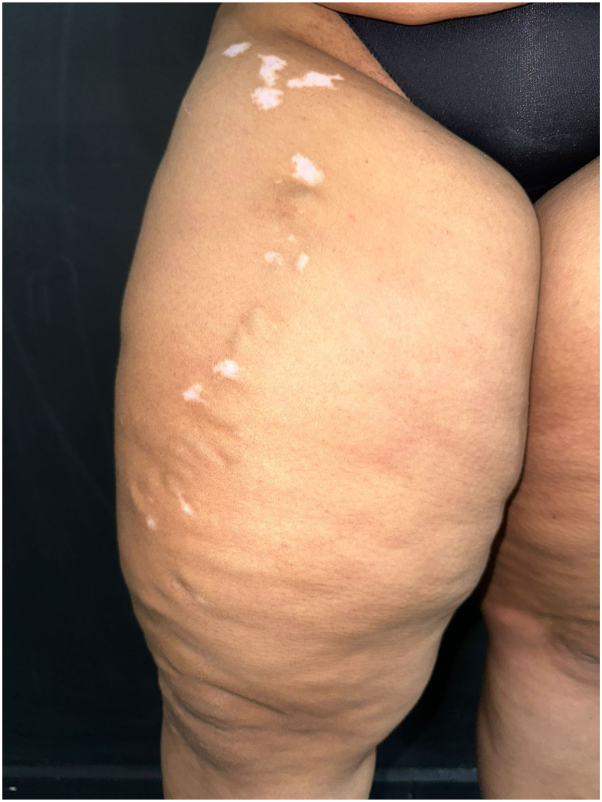
Scattered macules and patches of vitiligo overlying the dilated varix on the *right lower* extremity.

Case 2: A 36-year-old woman with a 5-year history of vitiligo presented to the dermatologist for follow-up. She had been treated with various topical corticosteroids without improvement. The patient denied any family history of vitiligo or other dermatologic diseases. The patient was in otherwise good health aside from an increased body mass index. Physical examination showed achromatic patches with islands of repigmentation on the face, trunk, and extremities. Patchy areas of dyspigmentation on the right upper thigh followed the course of the anterior accessory saphenous vein (Fig 2). The distended and tortuous appearance of this underlying vein was suggestive of koebnerizing generalized vitiligo. Treatment with 12 sessions of phototherapy, topical tacrolimus (0.1%, twice a day), and oral polypodium leucotomos (250 mg, every day) had resulted in improvement of the lesions.

**Fig 3:**
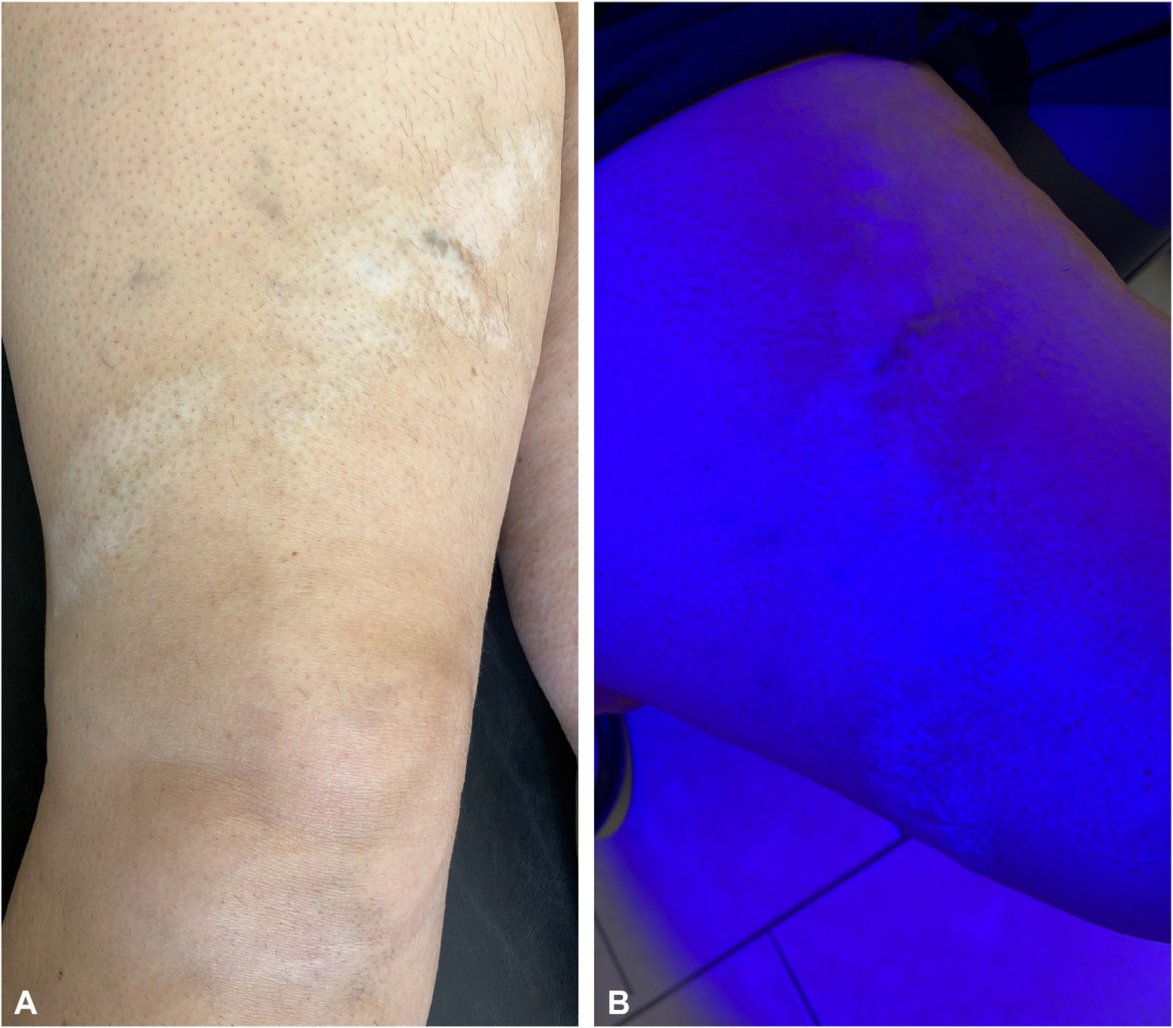
**A,** Linear patches of vitiligo on the *right upper* thigh. **B,** Wood’s lamp examination of vitiligo on the *right* ventral thigh.

Case 3: A 42-year-old female with a past medical history of hypothyroidism, hypertension, and a 20-year history of vitiligo presented to the dermatology clinic for follow-up. She had recently developed a linear patch of vitiligo on the right lower extremity (Fig 3, *A*). Upon further examination, the patient was noted to have noticeable varicose veins on the bilateral lower extremities. The patches of vitiligo grossly followed the pattern of a dilated varix on the right ventral thigh. Wood’s lamp examination of the area showed sharply demarcated blue-white patches (Fig 3, *B*), confirming the presence of vitiligo over the varicose vein.

## Discussion

Vitiligo affects approximately 1% of the world’s population, and the KP occurs in 21% to 62% of patients with vitiligo.^6^ Koebnerization has been documented to occur after trauma that is penetrating or mechanical in nature, such as stretching, compression, and friction.^1^^,^^3^^,^^4^ The KP is classified into 4 different categories, the first being ‘true koebnerization’, which occurs in psoriasis, vitiligo, and lichen planus.^1^^,^^3^^,^^4^^,^^6^ True koebnerization is secondary to many different forms of trauma, is reproducible, and is not due to infectious or allergic stimuli.^3^ Documented insults of causation include lacerations, skin grafts, tattoos, burns, radiation, incisions, gunshot injuries, electrodessication, freezing, tape stripping, nail manicures, shaving, and striae.^1^^,^^3^^,^^5^ Reports of koebnerization occurring solely after mechanical stimuli is comparatively more rare than when penetration injury to the epidermis has occurred, with some schools of thought believing epidermal injury to be a necessary component to the development of lesions.^3^^,^^4^ Others believe both the dermis and epidermis must experience some form of inflammation before koebnerization can occur.^5^ There is not yet a consensus on the exact pathogenesis behind KP, with genetics, hormones, infections, neural pathways, and enzymes all currently hypothesized contributors.^5^

In the present cases, vitiligo affected the skin over varicose veins in the 3 patients. None of the 3 patients presented received sclerotherapy or other procedures to treat their varicose veins, eliminating trauma from a surgical procedure as a source of the KP. Although coincidental segmental vitiligo is possible, the linear distribution over a dilated vein could be interpreted as the KP stimulated by skin stretching.^7^^,^^8^ The pathological adaptations of the vessel wall, such as an enlarged lumen diameter and remodeling of the extracellular matrix, may provide a mechanical stimulus above the necessary threshold to cause the overlying vitiligo.^9^ While the exact mechanism for varix-induced KP is yet to be established, the pathogenesis is likely multifactorial and involves alterations in dermal vascularity, loss of architecture of the collagen-elastin network in the vessel wall, excessive oxidative stress, deficient melanocyte growth factors, and a proinflammatory response leading to diminished adhesion molecules between melanocytes and keratinocytes.^4^^,^^6^^,^^9^

Most of the available literature regarding the KP stems from findings in psoriasis patients, limiting our current knowledge about the phenomenon’s significance.^3^ Once more, patients with vitiligo whom experience koebnerization react to different degrees after experiencing the same traumatic insult, and subsequently respond differently to the limited treatment options.^4^^,^^6^ It is known that koebnerization can occur at any cutaneous location and can affect patients at any point in their disease process.^5^ The affected location, intensity of trauma, and degree of lesion development greatly varies, however, providing a challenge for dermatologists in regards to informing patients of prevention strategies and prognostic factors.^4^ As the presence of koebnerization may indicate a poor therapeutic response and increased severity of disease, it is important for dermatologists to recognize potential rare presentations of the KP in their patients with vitiligo.^5^

## Conflicts of interest

None disclosed.
